# New Insights into Treating Early and Advanced Stage Diabetic Retinopathy

**DOI:** 10.3390/ijms23158513

**Published:** 2022-07-31

**Authors:** Rafael Simó, Cristina Hernández

**Affiliations:** 1 Diabetes and Metabolism Research Unit, Vall d’Hebron Research Institute (VHIR), Autonomous University of Barcelona, Vall d’Hebron Campus, 08035 Barcelona, Spain; 2 Centro de Investigación Biomédica en Red de Diabetes y Enfermedades Metabólicas Asociadas (CIBERDEM), Instituto de Salud Carlos III (ICSIII), 28029 Madrid, Spain

**Keywords:** diabetic retinopathy, diabetic macular edema, treatment, retinal neuroprotection, eye drops, intravitreal injections

## Abstract

Diabetic retinopathy (DR) is the leading cause of preventable blindness in the working-age population. The disease progresses slowly, and we can roughly differentiate two stages: early-stage (ESDR), in which there are mild retinal lesions and visual acuity is generally preserved, and advanced-stage (ASDR), in which the structural lesions are significant and visual acuity is compromised. At present, there are no specific treatments for ESDR and the current recommended action is to optimize metabolic control and maintain close control of blood pressure. However, in the coming years, it is foreseeable that therapeutic strategies based in neuroprotection will be introduced in the clinical arena. This means that screening aimed at identifying patients in whom neuroprotective treatment might be beneficial will be crucial. Regarding the treatment of ASDR, the current primary course is based on laser photocoagulation and intravitreal injections of anti-angiogenic factors or corticosteroids. Repeated intravitreal injections of anti-VEGF agents as the first-line treatment would be replaced by more cost-effective and personalized treatments based on the results of “liquid biopsies” of aqueous humor. Finally, topical administration (i.e., eye drops) of neuroprotective, anti-inflammatory and anti-angiogenic agents will represent a revolution in the treatment of DR in the coming decade. In this article, all these approaches and others will be critically discussed from a holistic perspective.

## 1. Introduction

Diabetic retinopathy (DR) is one of the most frequent chronic complications of diabetes and remains the leading cause of preventable blindness in the working-age population [1]. This statement has been recurrent in the introductions of articles and reviews on this issue from the last two decades, indicating that the advances in this field have not been sufficient for tackling the problem in an effective manner. In addition, given that diabetes is expected to increase from 537 million cases in 2011 to 643 million in 2030, DR will be an even more important problem in the near future [1]. In addition, it should be noted that DR is an independent predictor not only of microvascular but also macrovascular complications [2,3]. For instance, the presence of DR should be taken into account when evaluating the cardiovascular risk of a diabetic subject. Thus, the presence of DR has been associated with the development of macrovascular complications of diabetes, specifically, cerebrovascular complications, cardiovascular complications and vascular peripheral complications [4]. Moreover, the evaluation of retinal neurodegeneration could help to identify patients which are at risk of cognitive impairment, an emerging complication of the type 2 diabetes [5,6].

At present, DR is not considered only a microvascular disease, but rather, a complex complication in which neurodegeneration plays a significant role [7,8,9,10]. In fact, the American Diabetes Association (ADA) defines DR as a highly tissue-specific neurovascular complication [11]. Neuronal integrity is essential for vision, as it governs hue discrimination, contrast sensitivity, dark adaptation and visual fields, which are altered in a high proportion of patients in the early stages of the disease. These deficiencies are commonly imperceptible in daily life but have the potential to limit quality of life [12,13,14].

DR progresses slowly, and we can roughly differentiate two stages: early stage, in which there are mild retinal lesions and the visual acuity is generally preserved, and advanced-stage, in which structural lesions are significant and visual acuity is compromised [15]. This simple classification is useful when talking about therapeutic approaches, because only in advanced-stage DR can invasive treatments such as intravitreal injections be proposed. Early-stage comprises non-apparent DR as well as mild and moderate DR, whereas advanced-stage includes vision-threatening conditions such as severe non-proliferative (NPDR), proliferative DR (PDR) or severe diabetic macular edema (DME: the accumulation of excess fluid in the extracellular space within the retina in the macular area). Population-based studies have indicated that around about one third of the diabetic population have signs of DR, and approximately one-tenth are at risk of losing their vision, due to, e.g., DME or PDR [16].

The main pathogenic mechanisms involved in early-stage DR (ESDR) rely in the impairment of the neurovascular unit (NVU) and vasoregression of retinal capillaries [17,18]. NVU impairment includes glial activation and neurodegeneration of ganglion cells and photoreceptors, together with functional and structural alterations of endothelial cells and pericytes, which lead to the breakdown of the blood–retina barrier and cause vascular leakage. The vasoregression of capillaries contributes to hypoxia, which worsens neurodegeneration and triggers neovascularization. In advanced-stage DR (ASDR), the key pathogenic mechanisms are exaggerated vascular leakage, which results in diabetic macular edema (DME), and hypoxia/ischemia, which induces an imbalance between angiogenic and antiangiogenic factors, leading to neovascularizarion and so-called proliferative DR (PDR) [15]. Both DME and PDR are sight-threatening conditions. The main underlying molecular mechanisms for the development of DME are vascular endothelial growth factor (VEGF) and pro-inflammatory cytokines. VEGF is also a key pathogenic factor for PDR and, for this reason, it is the most important therapeutic target in advanced-stage DR.

**Figure 1:**
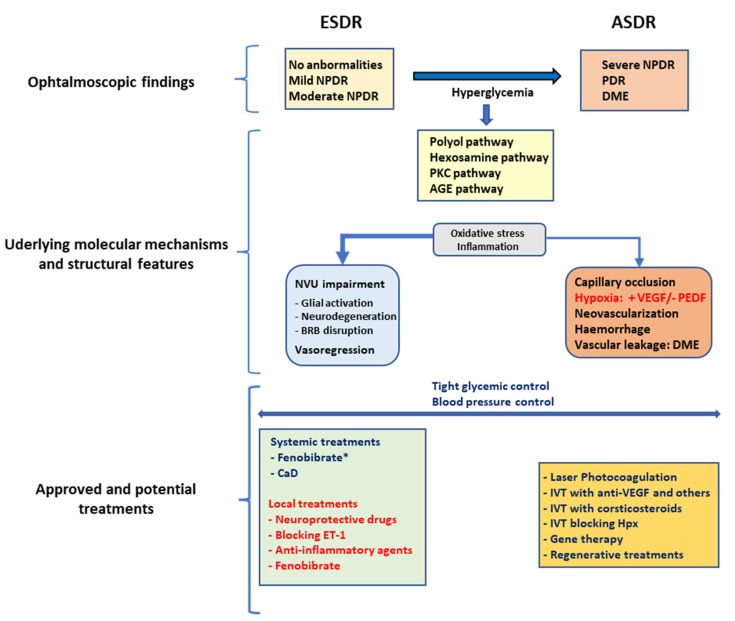
Schematic representation of the main ophthalmologic features of early and advanced stages of DR (ESDR and ASDR) and their main underlying mechanisms and structural features. The approved treatments (blue), and potential treatments (red) that are currently still in the preclinical development or in early clinical development. NVU: Neurovascular unit. IVT: Intravitreal treatment. (*) Fenofibrate has only been approved in few countries.

At present, there is no treatment for ESDR, and the current recommended action is to optimize blood glucose levels and lipid profile, and to keep blood pressure under control. By contrast, in ASDR, laser photocoagulation and/or intravitreal injections of corticosteroids or anti-VEGF agents are current treatments in clinical practice. However, these treatments are aggressive, expensive and cause significant adverse effects [9]. The main ophthalmologic features of early- and advanced-stage DR, their main underlying mechanisms and their structural features are schematically represented in Figure 1.

In this paper, a brief overview will be given of the advances in the experimental treatment of ESDR, as well as the main gaps to be filled. In addition, a critical review of current clinical practices for treating ASDR and new challenges will be provided.

## 2. New Insights for Treating ESDR

### 2.1. General Goals

Achieving glycemic control is a clear and well-founded form of treatment to prevent DR or arrest its progression [19,20,21]. Tight versus less tight glycemic control in the type 1 diabetic population of the Diabetes Control and Complications Trial (DCCT) reduced the risk of new retinopathy by 76% and of the progression of existing retinopathy by 54% [19]. Intensive vs. conventional glycemic management was associated with a 39% reduction in the risk of laser photocoagulation in the type 2 diabetic population of the UK Prospective Diabetes Study (UKPDS) [20].

Several randomized controlled trials (RCTs) have demonstrated the benefit of blood pressure (BP) control as a major modifiable factor for either incidence or progression of DR [22,23,24,25,26]. The UKPDS showed that after nine years of follow-up, the group under BP control had a 34% reduction in the risk of deterioration of DR, i.e., by two steps in the ETDRS scale (*p* = 0.0004), and a 47% reduction in the risk (*p* = 0.004) of deterioration in visual acuity, i.e., by three lines on the ETDRS chart [27]. Ambulatory blood pressure monitoring (ABPM) may provide better estimates of an individual’s average blood pressure and makes it possible to differentiate among several phenotypes [28]. Several of these phenotypes, including elevated mean 24-h BP, elevated night-time BP and non-dipping BP pattern, have been associated with increased cardiovascular risk [28,29,30,31]. However, no data regarding blood pressure phenotypes and the risk of DR have been reported.

Despite the influence of metabolic control and blood pressure on the development and progression of DR, there is clinical evidence that substantial variation exists. In fact, clinicians are aware that a subset of patients with poor control of glycemia and/or uncontrolled blood pressure does not develop DR. On the other hand, there are patients with good control of both blood glucose levels and hypertension that will develop DR. In fact, the Diabetes Control and Complications Trial/Epidemiology of Diabetes Interventions and Complications (DCCT/EDIC) Research Group showed that hemoglobin A1c (HbA1c) values explained up to 11% of the risk of DR, and that the unexplained 89% of variation in risk was due to elements of the diabetic milieu not captured by the mean HbA1c value [32].

At present there is no evidence that the prevention of hypoglycemic episodes influences the natural history of DR, and specific clinical studies aiming at addressing this important question are needed. This is particularly relevant from the general assumption that DR is a complex neurovascular disease in which neurodegeneration plays an essential role. The vulnerability of neurons faced with sustained and recurrent hypoglycemia is unquestionable, and therefore, hypoglycemia might lead to retinal neuron death.

Glycemic variability may also be involved in the weaknesses of HbA1c in predicting the development and progression of DR; studies showing that the reduction of glycemic variability can prevent DR or arrest its progression are needed [33,34,35]. Finally, changes in the epigenome may contribute to the phenomenon of “metabolic memory”, which explains the long-term effects of metabolic statuses obtained several years ago [36].

Dyslipidemia seems to have less influence than hyperglycemia or hypertension on the development and progression of proliferative DR or DME [37,38]. However, a growing body of evidence suggests that nontraditional lipid measures such as apolipoproteins A and B are stronger risk markers of DR than total cholesterol and triglyceride levels [39,40].

Heritability of DR has been estimated to be around 27% for DR and 52% for PDR. Among the large number of putative genes and genetic variants reported in the literature, ALR2, VEGF and RAGE exhibit consistent associations with DR. However, a huge effort to validate these results in multiple populations is needed before we can use these genes as biomarkers to indicate high risk [41]. In addition, specific gene variants in ICAM1, PPARGC1A and MTHFR have been associated with different NDPR phenotypes [42]. These results support the concept that different pathogenic mechanisms are involved in the different risks of progression of NDPR phenotypes. However, further confirmation in larger cohorts is needed.

### 2.2. Systemic Treatments

There is evidence based on clinical trials that fenofibrate, a peroxisome proliferator-activated receptor alpha (PPARα) currently used as a hypolipidemic agent, slows the progression of DR and reduces the need for laser photocoagulation and vitrectomy surgery [43,44,45]. However, the progression of DR was not the primary outcome of these clinical trials, and the scientific community has not accepted fenofibrate as an established treatment for arresting the progression of DR. To fill this scientific gap, a large randomized clinical trial is ongoing in the US (ClinicalTrials.gov Identifier: NCT04661358). It should be noted that the multifaceted non-lipidic actions of fenofibrate seem more important in reducing the progression of DR than the lipidic-mediated mechanisms [46].

Calcium dobesilate (CaD) is a safe drug that has been approved for many years for the treatment of DR in a large number of countries. However, it has not been broadly used in clinical practice. Two randomized placebo-controlled trials demonstrated the effectiveness of CaD in preventing the progression of early-stage DR [47,48], but its effectiveness in AEDR remains to be determined. As occurs with fenofibrate, CaD targets multiple pathogenic pathways involved in DR [49]. However, further research to better understand the mechanisms of action and more targeted clinical trials are needed.

Some clinical trials have highlighted renin-angiotensin system (RAS) blockade as a promising systemic treatment for DR [9]. However, there is no robust clinical evidence on this issue. In fact, two large clinical trials, the Diabetic Retinopathy Candesartan Trials (DIRECT) program [50,51] and the Action in Diabetes and Vascular Disease (ADVANCE) study [52], failed to demonstrate any beneficial effect, taking into account the primary endpoints. Therefore, the classic concept that lowering blood pressure is the most important strategy, regardless of the choice of drug, has re-emerged with renewed rigor.

There is no evidence that any given anti-diabetic treatment could exert a beneficial effect on DR onset or progression unrelated to the improvement of blood glucose levels. However, when evaluating a diabetic subject, knowledge about the presence and degree of DR has therapeutic implications. In this regard, there is mounting evidence that the rapid control of blood glucose levels can worsen DR [53]. This is a well-recognized effect in ASDR but is less evident in ESDR, and therefore, will be further detailed below.

### 2.3. Targeting NVU

NVU comprise diverse neural cell types (i.e., amacrine cells, ganglion cells, horizontal and bipolar cells), glia (astrocytes and Müller cells), professional immune cells (microglia and perivascular macrophages) and vascular cells (endothelial cells and pericytes) [17]. In recent years, several experimental approaches attempting to prevent diabetes-induced NVU impairment have been reported, but long-term clinical trials to support this therapeutic strategy in the context of DR are needed.

Route of administration is an issue in ESDR, because vision is generally preserved and repeated intravitreal injections represent an aggressive and unacceptable treatment. The systemic administration of drugs to block the main pathogenic pathways involved in DR has two main problems. First, the blood–retinal barrier impedes their reaching the retina at pharmacological concentrations; and second, systemic administration could lead to adverse effects and pharmacologic interference with other drugs used for the treatment of diabetes and its co-morbidities. Eye drops containing small molecules have been shown to be effective in treating early-stage DR in experimental models [54]; such compounds can reach the retina by the trans-scleral route [55,56,57]. In addition, topical administration limits the action of such compounds in the eye and minimizes the associated systemic effects.

Several proteins with neurotrophic activity that are required for retinal homeostasis are synthesized by the retina. However, in the diabetic retina, a downregulation exists of these neurotrophic factors. As such, a therapeutic strategy based on their repalcement seems resonable. In fact, treatments using eye drops seeking to replace these natural neuroprotective factors, such as pigment epithelial growth factor (PEDF), somatostatin and glucagon-like peptide 1 (GLP-1), or to avoid their degradation, such as DPP-IV inhibitors to enhance GLP-1, have been effective in experimental models [55,56,58,59,60]. Apart from neuroprotection, PEDF, GLP-1 and DPP-IV inhibitors significantly abrogate vascular leakage and, therefore, can be envisaged as promising treatments which should be tested in the clinical arena.

The blockade of endothelin-1 is also a very interesting approach. ET-1 and its receptors (ETA-R and ETB-R) are upregulated in ESDR [61,62,63]. The activation of ETA-R mainly mediates vasoconstriction and vasoregression [64], while the activation of ETB-R induces retinal neurodegeneration [64,65]. Topical (eye drops) administration of Bosentan, by blocking ETA-R and ETB-R, exerted a beneficial effect on both the microvasculature and neurons and prevented retinal neurodegeneration and vascular leakage in db/db mice [57]. These promising results suggest that therapeutic strategies based on blocking ET-1 should be tested in clinical trials.

Treatments based on targeting inflammation are an emerging field and several approaches, such as the blockade of IL1β [66] or TNFα [67,68,69,70], or the stimulation of suppressors of cytokine signaling (SOCS) [71], have had successful results in experimental research. In addition, the systemic administration of salicilates, and the inhibition of several mediators involved in inflammatory responses such as COX-2 and NFkB, have proved useful in reducing DR in experimental models [72]. However, clinical evidence of the efficacy of anti-inflammatory treatments, i.e., either eye drops or systemic administration, is lacking, and further research on this issue is required.

## 3. Advances for Treating ASDR

### 3.1. General Measures

Tight control of blood glucose levels and blood pressure is recommended in ASDR, but the beneficial effects are less evident than when these general measures are implemented at the beginning of the disease or when ESDR appears.

The therapeutic goal of tight metabolic control should be balanced against the risk of hypoglycaemia, especially in older people, in whom aggressive glycaemic control does not further reduce retinopathy risk, and might even be associated with increased mortality [73]. In addition, an initial worsening of DR has been reported as a consequence of the rapid improvement of hyperglycemia in both type 1 and type 2 diabetes [74,75,76,77]. The most important risk factors for early worsening were shown to be a higher hemoglobin A1c level at baseline, a large reduction of HbA1c (>2%) and the severity of DR at baseline [78,79,80]. Recently, systematic reviews confirmed this concept and suggested that early worsening of DR could be particularly relevant when HbA1c is reduced to >1.5% after 3 months or to >2% after 6 months [81].

### 3.2. Critical Overview of Current Treatment

The treatment for ASDR is based on laser photocoagulation and intravitreal injections of anti-angiogenic factors or corticosteroids. Fenofibrate seems to be a reasonable treatment to arrest progression to sight-threatening DR but, as previously mentioned, a specific clinical trial supporting this indication is still lacking. Vitreo-retinal surgery is a complex and expensive treatment that is reserved for the ultimately blinding complications of DR [82].

#### 3.2.1. Laser Treatment

Laser photocoagulation is generally indicated in PDR or in clinically significant DME and prevents further deterioration of vision if applied sufficiently early in the progression of the disease [83]. However, laser treatment does not usually restore lost vision and is associated with potentially severe adverse effects such as impaired adaptation to changes in light levels, some loss of visual acuity, loss of peripheral vision, changes in colour vision and exacerbation of macular edema.

#### 3.2.2. Anti-Angiogenic Treatment

Intravitreal anti-VEGF agents are currently used as a first-line treatment for clinically significant DME [84]. Although both proinflammatory cytokines and VEGF play a crucial role in the pathogenesis of DME, the blockade of VEGF, rather than an anti-inflammatory approach, is primarily used [85]. Clinical trials have provided robust evidence that the intravitreous administration of anti-VEGF agents is better than laser therapy both at preserving and improving the vision of patients with DME [15,84,85]. However, around 50% of DME patients do not adequately respond to anti-VEGF therapy [86]. These findings support the concept that other mechanistic pathways may operate independently or in conjunction with VEGF in the pathogenesis of ASDR. In this regard, in the seminal study by Aiello et al. [87], 36% of the patients with PDR did not show increased VEGF levels in the vitreous fluid. Therefore, it was not surprising that a high rate patients showed an inadequate response to anti-VEGF treatment. In this subgroup of non-responders, proinflammatory cytokines/chemochines and other angiogenic factors unrelated to VEGF (platelet-derived growth factor, basic fibroblast growth factor, hepatocyte growth factor, angiopoietin-2) probably play a more relevant pathogenic role.

Aflibercept produces a more extensive angiogenic blockade than anti-VEGF agents and may provide superior outcomes in certain patients compared with treatments that inhibit only VEGF (i.e., bevacizumab and ranibizumab) [88]. Aflibercept is a soluble decoy receptor that binds vascular endothelial growth factor-A (VEGF-A), VEGF-B and placental growth factor (PIGF) with a greater affinity than the body’s native receptors. Therefore, VEGF binds to aflibercept instead of its original receptors, thereby reducing the activity of VEGF [89].

A potential serious complication of pan-VEGF inhibition is the neurodegeneration of the remaining healthy retina [90,91,92]. This can occur because VEGF is a powerful neurotrophic factor, raising questions about the potential use of neuroprotective agents to prevent this adverse effect. Specific studies seeking to address this important question are warranted.

The angiopoietin (Ang)/Tie2 signalling axis, a key regulator of angiogenesis, has emerged as a potential therapeutic strategy, and several clinical trials have demonstrated the efficacy of pharmacologic and biologic mediators of the Ang/Tie2 pathway [93]. The VEGF pathway is important for inducing endothelial cell sprouting and primary network formation, whereas the Ang/Tie2 pathway regulates blood vessel remodelling and maturation in the later stages of the angiogenic process [94]. The simultaneous inhibition of angiopoietin-2 and VEGF-A with faracimab have been shown to be superior to anti-VEGF alone at week 24 in treatment-naive patients with DME [95].

#### 3.2.3. Corticosteroids

Intravitreal corticosteroids are mainly used due to their anti-inflammatory mechanisms. Intravitreal use limits the action to the eye and makes it possible to reach pharmacological concentrations. However, the complication rate (in the eye) of intravitreal corticosteroid injections is high, e.g., glaucoma and cataract formation [96]. For this reason, the use of intravitreal corticosteroids is generally restricted to patients affected by persistent or refractory DME, especially in pseudophakic eyes [97]. Apart from abrogating the effects of proinflammatory cytokines, corticosteroids are able to exert neuroprotection, as has been proven in experimental [98] and clinical studies [99]. Furthermore, they also have antiangiogenic action [100]. These multifaceted effects have led to increased interest in these classic drugs, and sustained release formulations or implants that reduce the frequency of intravitreal injections are now available [96].

## 4. Discussion

The question of who should receive neuroprotective agents is a matter of debate in the treatment of ESDR. The results from the EUROCONDOR study provided evidence that a significant proportion of individuals (35%) with type 2 diabetes present with early microvascular disease without detectable neurodysfunction [101,102]. Therefore, neurodegeneration is not always the apparent primary event in the natural history of DR. In this regard, it is possible that neurodegeneration could herald DR in some patients, but that neurodegeneration and microvascular disease could occur independently in others. On this basis, the screening for neurodegeneration assessed by function (i.e., mfERG (with or without flickering), microperimetry) or morphology (i.e., SD-OCT) will be critical in terms of identifying patients which might benefit from neuroprotective treatment. Another question is whether neuroprotection should be implemented in patients with neurodysfunction or neurodegeneration without any visible microvascular abnormality. Since neuron loss is related to deficient sensory capacity and vision-related quality of life, it seems reasonable to treat neurodysfunction or neurodegeneration per se as a specific target. Periodic assessments of the effectiveness of the selected neuroprotective agent would be recommended. Finally, nanotechnology can further improve the delivery of drugs by topical administration, and it is foreseeable that a myriad of neuroprotective drugs will reach the market in the near future.

More cost-effective and personalized treatments are needed for AESD. Although intravitreal injections are very safe and effective, they require frequent monitoring and repeated visits for imaging, examinations and injections over a long period of time. Therefore, they constitute a significant burden on patients, ophthalmologists and health systems. In addition, as previously stated, intravitreal anti-VEGF treatments fail in a significant proportion of patients, revealing that VEGF unrelated pathways play a primary role in these patients. The sampling of aqueous humor during the first injection (“liquid biopsy”) could be useful for examining the predominant pathogenic pathway and would permit us to select a more rational and (probably) more cost-effective treatment [103]. The identification of other players in the pathogenesis of DME and PDR will extend the therapeutic options. In this regard, the potential role of hemopexin (Hpx), which is an abundant protein in the vitreous fluid of diabetic patients with DME, is worth mentioning [104]. It should be noted that Hpx is the best characterized permeability factor in steroid-sensitive nephrotic syndrome, and its infusion induces experimental proteinuria [105,106]. Therefore, it is possible that it also contributes to hyperpermeability at the blood–retinal barrier. In fact, we have shown that Hpx, within the same range as that detected in the vitreous fluid of patients with DME (50 μg/mL), leads to the disruption of retinal pigment epithelium (RPE) cells (outer blood-retinal barrier), thus increasing permeability, and that this effect is prevented by pretreatment with anti-Hpx antibodies [107].

The efficacy of the topical administration (i.e., eye drops) of neuroprotective agents aimed at improving vision-related quality of life in patients with ASDR, as well as their role in mitigating the neurodegeneration associated with long-term anti-VEGF blockade, need to be clarified. In addition, the potential effectiveness of topical administrations of antiangiogenic agents instead of intravitreal injections should also be examined. It is noteworthy that the successful therapeutic effects of nano-eye drops containing fenofibrate on neurovascular coupling, vascular leakage and neovascularization have been recently reported in experimental models of diabetes [108,109].

The identification of patients in whom neurodegeneration plays a key role in the development and progression of DR is a challenge. The identification of this specific phenotype will be crucial for selecting neuroprotective drugs and the implementation of more personalized and cost-effective treatments. Progress in the development of new technologies in retinal imaging will speed up progress in this respect.

One limiting factor that has hampered the investigation of new drugs for DR is the low sensitive endpoints in clinical trials, which require large sample sizes as well as a long duration to achieve a statistical power which is sufficient to determine treatment effectiveness. Thus, a critical point is to change the metrics used in the assessment of clinical trials based on the current regulatory framework (ex. 2–3 steps of progression in the ETDRS scale). New methods of retinal assessment, such as angio-OCT, could revolutionize the design of clinical trials aimed at testing new drugs for DR treatment, i.e., through a significant reduction in the required sample size and by shortening the required follow up time.

In summary, progress in the development, testing and implementation of new therapeutic approaches for DR requires the involvement of multiple stakeholders including scientists, clinicians, regulatory agencies and patients. This will allow us to design rational and cost-effective strategies to treat this frequent and resource-consuming complication of diabetes.
